# Population Genetic Structure of *Chlorops oryzae* (Diptera, Chloropidae) in China

**DOI:** 10.3390/insects13040327

**Published:** 2022-03-25

**Authors:** Xianya Li, Shunjiao Wu, Yonghong Xu, Yinghong Liu, Jia Wang

**Affiliations:** College of Plant Protection, Southwest University, Chongqing 400715, China; xianya_li@126.com (X.L.); wushunjiao0130@126.com (S.W.); xyh202202@126.com (Y.X.)

**Keywords:** *C. oryzae*, COI, ITS1, genetic differentiation, genetic diversity

## Abstract

**Simple Summary:**

Recently, *Chlorops oryzae* has become one of the major pests of rice in some regions, which has caused serious economic losses. To understand the genetic mechanisms of frequent local outbreaks and population expansion of *C. oryzae*, we analyzed the population genetic structure using two molecular markers, COI and ITS1 sequences. The results indicated that the *C. oryzae* populations experienced rapid expansion after a “Bottleneck effect” and the local outbreaks were probably caused by frequent gene flow among populations.

**Abstract:**

Frequent outbreaks have made *Chlorops oryzae* one of the major pests of rice in some regions. In order to understand the ecological adaptation of *C. oryzae* at the molecular level, and provide a scientific basis for formulating management strategies, we used two molecular markers, COI and ITS1 sequences, to systematically analyze the genetic structure of 31 populations. The higher haplotype diversity and lower nucleotide diversity indicated that the *C. oryzae* populations experienced rapid expansion after a “Bottleneck effect”. The results of the mismatch distribution, neutrality test (Fu’s Fs < 0, *p* < 0.001), and haplotype network analysis suggested that the population has recently undergone an expansion. Although genetic differentiation among *C. oryzae* populations was found to have existed at low/medium levels (Fst: 0.183 for COI, 0.065 for ITS1), the frequent gene flow presented as well (Nm: 2.23 for COI, 3.60 for ITS1) was supposed to be responsible for frequent local outbreaks.

## 1. Introduction

Population genetic structure, the most basic genetic information of a species, represents the amount and distribution of genetic variation within and among populations. Moreover, it is the accumulation of the evolutionary history and the basis for the development of future evolutionary adaptations of a species [1,2]. Population genetic structure is usually indicated with genetic diversity, genetic differentiation, and genetic distance [3]. Analysis of population genetic structure is conducive to revealing the extent and pattern of gene flow and establishing phylogenetic relationships among populations, thereby contributing to understanding population dynamics, occurrence trends, and genetic relationships among populations [4,5].

Molecular markers are a direct reflection of genetic diversity, which is a very effective tool to study the genetic structure of species [6]. Presently, molecular markers, such as mitochondrial DNA (mtDNA) [7], ribosomal DNA (rDNA) [8], and microsatellites [9], have been widely used in the analysis of the genetic structure of insect populations. As one of the classical molecular markers, mtDNA supports a better understanding of the process of population dispersal and evolution because of its small size, high level of mutations, strict adherence to matrilineal inheritance, lack of introns, and recombination characteristics [10]. Particularly, the mtDNA cytochrome oxidase I (COI) gene has been widely used owing to its moderate evolutionary rate and readily available advantages [11,12,13,14]. However, the genetic diversity and evolutionary history of mtDNA are not necessarily identical to, or representative of, the organism due to potential introgression, lower effective population size, and potential selection. In light of this, combining mtDNA and other markers can greatly improve the reliability of results [15]. For example, the population dispersal pathways and timing of *Meromyza saltatrix* were studied in connection with the COI gene and morphological features [16,17]. Moreover, nuclear genes (nDNA) which have genetic information that mtDNA lacks are able to play a complementary role to mtDNA. The rDNA internal transcribed spacer 1 (ITS1), a non-coding sequence located between 18S and 5.8S rDNA with high nucleotide polymorphism in most eukaryotes, has also been widely used as markers to study population genetic structure [18]. For example, the genetic structure of 13 geographic populations of *Melipona subnitida* in northeastern Brazil was analyzed using ITS1 sequences [19,20]. The analysis of genetic diversity of invasive species *Halyomorpha halys* in North America and Europe using ITS1 and COI markers suggested that the joint use of ITS1 and COI could improve the accuracy of detection of the source areas of an invasion [21].

*Chlorops oryzae* (Diptera, Chloropidae), an important rice pest, is widely distributed in Asia, such as China, Japan, and Korea [22]. The larvae of *C. oryzae* bore into the stem and move to the growing point, where they feed on the developing leaves and young panicles, inhibiting the effectiveness of chemical insecticide [23,24]. In recent years, with changes in agroecology, cultivation and farming system, climate, and control agents, the *C. oryzae* occurrence areas have been expanding, and the damage levels are geographically different [25]. The frequent outbreaks have made *C. oryzae* one of the major pests of rice in some areas [26], causing yield losses of 20–50% [27]. In China, *C. oryzae* occurs 2–5 generations per year in different regions, mainly dependent on diapause induction and duration [23]. Presently, studies on *C. oryzae* are still limited and have mainly focused on physiology and ecology [28,29]. Till recently, Zhou et al. [30] used COI and ISSR markers to analyze its genetic structure and speculated that frequent gene flow in *C. oryzae* populations was responsible for outbreaks. However, the genetic mechanisms underlying different occurrence levels and gradual geographic expansions are still unknown.

In this study, we used two molecular markers, COI and ITS1 sequences, to systematically analyze the genetic structure of 31 geographic populations collected from the main distribution areas of *C. oryzae* in China. The results of this study will contribute to understanding the ecological adaptation of *C. oryzae* at the molecular level, thereby providing a scientific basis for formulating management strategies.

## 2. Methods

### 2.1. Sample Collection and DNA Extraction

**Figure 1:**
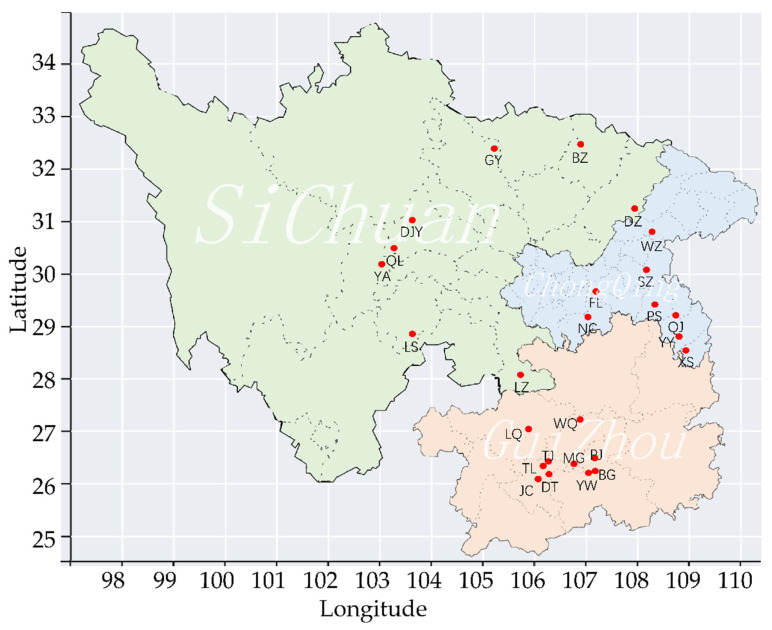
Collection sites of populations. Populations data for TY, ZZ, LH, XT, HX, and ZJ were obtained from Zhou et al. [30] and not marked in the figure.

Samples of 26 *C. oryzae* populations were collected from Guizhou, Chongqing, and Sichuan Provinces, China, from May 2020–August 2021 (Figure 1 and Table S1). All samples were soaked in 75% ethanol and stored at −20 °C until total DNA extraction. Genomic DNA was extracted according to the method of DeBarro and Driver with minor modification [31]. Briefly, samples were soaked in deionized water to remove alcohol prior to extraction. Individual samples were grounded thoroughly in centrifuge tubes with 30 μL of the lysis buffer (50 mM KCl, 10 mM Tris pH 8.4, 0.45% Tween 20, 0.2% gelatin, 0.45% NP40, 60 μg/mL proteinase K) to form a homogenate, which was incubated at 65 °C for 30 min and then boiled for 10 min to inactivate the proteinase K. Total DNA extraction were stored at −20 °C for subsequent analysis.

### 2.2. PCR Amplification and Sequencing

The genomic DNA extracted from *C. oryzae* was used as a template for COI and ITS1 amplification. The COI fragments were amplified with specific primers COI F (5′-CTA GGT GCT CCA GAT ATA GCA TTT C-3′) and COI R (5′-GGC TAA AAC AAC TCC TGT TAA TCC-3′) [30]. The ITS1 fragments were amplified with primers ITS1 F (5′-CGC ATT ATG TGT TAC GGA TGT T-3′) and ITS1 R (5′-GGT TGC GAA TGT CTC TAA TTC-3′). PCR was performed in 30 μL volumes comprised of 15 μL 2 × Taq PCR MasterMix (Biomed, Beijing, China), 1 μL of each primer (10 mmol/L), 1 μL of template DNA solution, and 12 μL double distilled water. Amplifications were conducted as follows: 34 cycles of denaturation at 94 °C for 30 s, annealing at 55 °C for 30 s, and extension at 72 °C for 1 min. All PCR products were checked by electrophoresis on a 1% agarose gel and bi-directionally sequenced by Sangon Biotech (Shanghai, China). Sequences were deposited in the GenBank under accession numbers OM490688-OM491162 for COI and OM540945-OM541304 for ITS1. COI sequence data of six populations (Hunan: TY, ZZ, LH, XT; Guizhou: HX; Zhejiang: ZJ) were kindly provided by Zhou et al. [30].

### 2.3. Data Analysis

The sequencing data were edited using SnapGene v.4.2 [32]. All data processing, including basic statistics and calculation of inter- and intraspecific genetic distances (Kimura 2-parameter model for COI and Tamura 3-parameter model for ITS1) and transition/transversion (ts/tv) ratio, were performed using MEGA v.7.0 [33].

The number of haplotypes (h), haplotype diversity (Hd), average number of nucleotide differences (k), nucleotide diversity (Pi), and haplotype analysis were calculated by DnaSP v.5.10 [34]. Neutrality tests (Fu’s Fs [35] and Tajima’s D [36]), F-Statistics (Fst) (Bonferroni correction for significance), gene flow (Nm) (COI: Nm = (1 - Fst)/2Fst; ITS1: Nm = (1 - Fst)/4Fst), and analysis of molecular variance (AMOVA) were performed using Arlequin v.3.15 [37]. The distribution of pairwise differences between individual sequences was analyzed by mismatch distribution analysis using DnaSP. In addition, the statistics of the raggedness (*rg*) index of the observed distribution and the sum of square deviations (SSD) between the observed and the expected mismatch were also calculated using Arlequin based on the spatial expansion model. The statistical significance of variance components in Arlequin was tested with 1000 permutations.

Geographical distances among populations were calculated using MapInfo Professional v.8.5 (Table S2) [38]. Mantel test was conducted by NTsyspc v.2.1 for the natural logarithm of interspecific genetic distance (Fst/(1 - Fst)) and geographic distance [39]. The haplotype networks were constructed using the median-joining method in software Network v.10.2 [40]. Compared with traditional phylogenetic trees, haplotype network diagrams can better reveal the genealogical relationships between conspecifics.

## 3. Result

### 3.1. Base Composition and Gene Mutation

A total of 598 COI sequences (475 obtained in this study and 123 from Zhou et al. [30]) representing 31 populations and 360 ITS1 sequences representing 26 populations were used for subsequent analysis (Table S1). The final aligned COI sequence fragments were 720 bp and all alignments were unambiguous, with no insertions or deletions. The average nucleotide composition was T = 36.4%, C = 17.4%, A = 29.6%, and G = 16.6%, showing an obvious AT bias (66.0%). In total, 57 polymorphic sites (7.92%) were detected in all COI sequences, including 34 singleton variable sites and 23 parsimony informative sites. There were 47 transitions and 10 transversions, and the overall ts/tv bias was 6.934. The ts/tv rate ratio was observed to be higher with purines (20.78) than pyrimidines (11.08).

The final aligned ITS1 sequence fragments were 607 bp. The average nucleotide composition was T = 31.2%, C = 14.8%, A = 36.5%, G = 17.5% and A + T = 67.7%. All sequences had a total of 20 polymorphic sites (3.29%), including 10 single variable sites and 10 parsimony informative sites. There were 8 transitions and 12 transversions, and the overall ts/tv bias was 0.639. The ts/tv rate ratio was observed to be higher with purines (2.402) than pyrimidines (0.116).

### 3.2. Genetic Diversity

For COI analysis, the 31 *C. oryzae* populations had a total of 55 haplotypes, with an overall Hd of 0.346, k of 0.854, and Pi of 0.0012. The Hd, k, and Pi of each population ranged from 0.000–0.638, 0.000–3.985, and 0.0000–0.0052, respectively. LZ and NC populations had higher levels of genetic diversity (Hd > 0.5, Pi > 0.005) (Table 1).

For ITS1 analysis, the 26 *C. oryzae* populations had a total of 26 haplotypes, with an overall Hd of 0.750, k of 1.551, and Pi of 0.0026. The Hd, k, and Pi of each population ranged from 0.400–0.956, 0.400–2.911, and 0.0007–0.0048, respectively. Except for the YW and SZ populations, all populations had higher levels of Hd (Hd < 0.5) and lower levels of Pi (Pi < 0.005) (Table 2).

### 3.3. Population Demographic History

**Figure 2:**
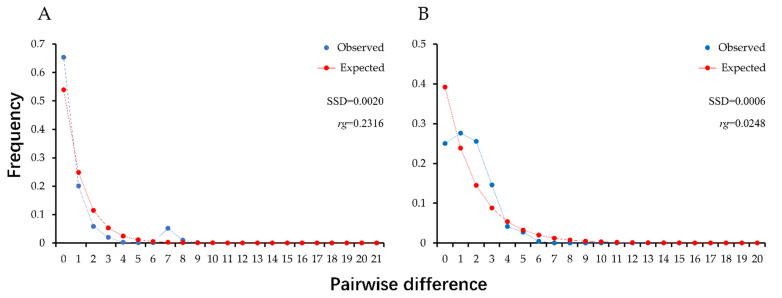
Mismatch distribution of pairwise nucleotide differences for all populations of *C. oryzae* based on COI sequence (**A**) and ITS1 sequence (**B**). (SSD) Sum of squared deviation; (*rg*) raggedness index.

When all samples were taken as one population, the neutrality test and mismatch analysis of the *C. oryzae* population were performed based on COI and ITS1 sequences. The Tajima’s D and Fu’s Fs values of the total population were all negative, and all Fu’s Fs values were significant (*p* < 0.01) (Table 1 and Table 2). Besides, the mismatch distribution of both markers in the total populations showed a single-peaked form, indicating that the population experienced expansion events. In addition, both the statistical reference SSD and *rg* values did not reach significant level, supporting the spatial expansion model (COI: SSD = 0.0020, *p* = 0.7000, *rg* = 0.2316, *p* = 0.8000; ITS1: SSD = 0.0006, *p* = 0.8730, *rg* = 0.0248, *p* = 0.9050) (Figure 2).

### 3.4. Genetic Differentiation

**Figure 3:**
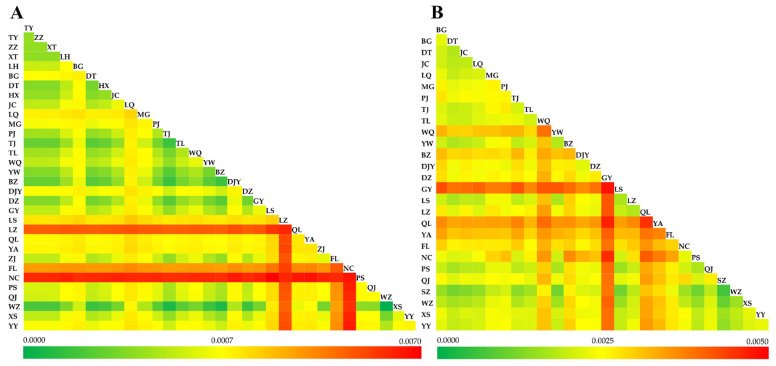
Genetic distances between and within *C. oryzae* populations based on COI sequences using the Kimura’s 2-parameter model (**A**), and ITS1 sequences using the Tamura 3-parameter model (**B**).

Genetic distances within and between populations were estimated based on COI and ITS1 sequences. For COI analysis, the inter- and intra-population genetic distance ranged from 0.0001–0.0060 and 0.0000–0.0056, respectively (Figure 3A), indicating that genetic distances between populations were higher than those within populations. For ITS1 analysis, the two values ranged from 0.0010–0.0047 and 0.0007–0.0048, respectively (Figure 3B).

The results of AMOVA suggested that the genetic variation in *C. oryzae* populations was mainly from within populations, while less from among populations (Va < Vb, *p* < 0.001). Moreover, there was some degree of genetic variation within the overall populations (Fst > 0.05, *p* < 0.001) (Table 3).

The Fst and Nm values between pairwise populations were calculated based on COI and ITS1 sequences (Table 4 and Table 5). The Fst values ranged from 0.000–0.536 and 0.000–0.307 for COI and ITS1 sequences, respectively. For COI analysis, the genetic differentiation was mainly attributed to individuals in the NC population (Fst > 0.25, Nm < 1). A three-level AMOVA analysis of the NC population with the rest of the populations based on COI markers showed the presence of significant genetic differentiation (Fst = 0.69, *p* < 0.001).

**Figure 4:**
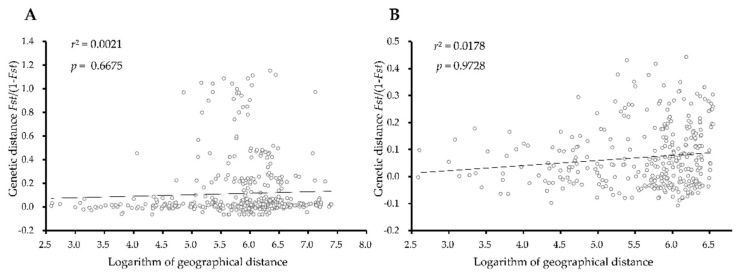
Relationship between genetic distance (*Fst*/(1 - *Fst*)) and logarithm geographic distance of *C. oryzae* populations based on COI sequence (**A**) and ITS1 sequence (**B**).

The Mantel tests based on both markers did not support a significant correlation between geographic distance and genetic distance, thus excluding the effect of distance segregation on genetic differentiation (Figure 4).

### 3.5. Haplotype Network Analysis

**Figure 5:**
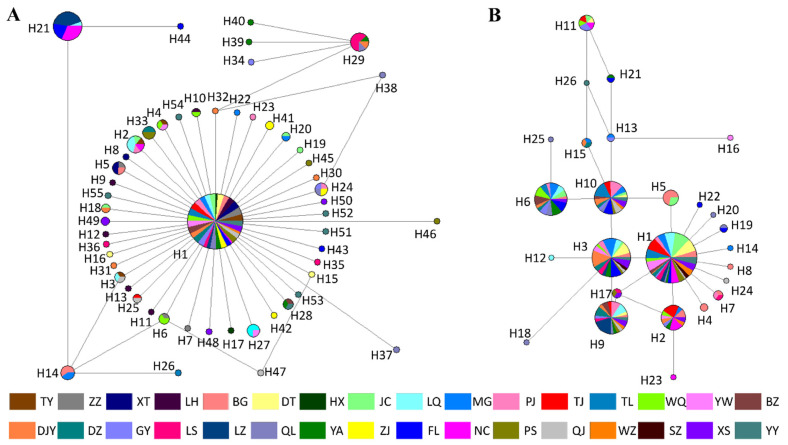
Median-joining network of haplotypes for *C. oryzae* based on COI haplotypes (**A**) and ITS1 haplotypes (**B**). Each circle represents a haplotype, and the area of a circle is proportional to the number of individuals with that haplotype. Colors within nodes refer to *C. oryzae* sampling regions.

To understand the relationships of identified haplotypes, the median-joining haplotype network was constructed (Figure 5). Among the 55 COI haplotypes, H1 occupied the center of the network and was shared by all populations. H1 was also the most common haplotype, accounting for 80.6% of all samples. The remaining 54 haplotypes were distributed around H1 in a star pattern. H21 and H44 were far away from H1 and mainly shared by NC, LZ, and FL populations (Figure 5A). Among the 26 ITS1 haplotypes, H1 was the ancestral haplotype shared by all populations and occupied a central position in the network. The four haplotypes, H3, H6, H9, and H10, were derived from H1 and collectively accounted for 37.5% of all samples (Figure 5B).

## 4. Discussion

Genetic diversity, which refers to the sum of genetic variation among populations within a species or individuals within a population [1], is a fundamental guarantee for maintaining species evolution [2]. Genetic diversity is caused by the variation of genetic material and is influenced by mutation rate, effective population size, gene flow, and other factors [41]. A population with rich genetic diversity often possesses a strong adaptability to the environment, thus facilitating population outbreaks and their large-scale spread.

Haplotype diversity and nucleotide diversity are the main indicators of genetic diversity. In this study, the ITS1 analysis presented that *C. oryzae* populations had higher haplotype diversity (Hd > 0.5) and lower nucleotide diversity (Pi < 0.005), suggesting that the population experienced a recent “Bottleneck effect” followed by a short period of rapid population expansion [42]. Meanwhile, the COI analysis revealed low haplotype diversity, probably due to different genetic patterns of molecular markers [43]. This is consistent with the finding that ITS1 possesses greater genetic diversity than COI in *Halyomorpha halys* [21].

Historical population demographics is one of the core elements of molecular phylogeography. The analysis of historical population demographics is in favor of understanding the effects of external environmental factors on population development and distribution, and also provides a reference for developing pest management strategies [44]. To this end, neutrality tests (Tajima’s D and Fu’s Fs values) and mismatch distributions are commonly used [45,46]. In this study, when all samples were calculated as a population, the Tajima’s D and Fu’s Fs values were significantly negative except for Tajima’s D for ITS1 data, indicating the expansion of the population size, which is consistent with the previous speculation [25]. Additionally, the extremely small Fu’s Fs values (−3.40 × 10^38^) implied that the population expansion occurred not so long ago as the Fu’s Fs values are more sensitive to recent population expansion [35]. Likewise, the mismatch distribution showed population expansion as well because of the single-peaked curve, insignificant SSD values, and small *rg* values [46]. Moreover, the haplotype network for COI with a star-shaped distribution further supported speculation of population expansion [47].

Genetic distance, AMOVA [48], Fst value [49], and Nm value [50], are important indicators of the genetic differentiation of populations. The genetic distance between populations was generally close except for NC and LZ for COI, and the genetic variation was mainly from within the population revealed by AMOVA. Similarly, the Fst and Nm values between paired populations indicated that the genetic differentiation between populations was stemmed from a few populations such as NC. In fact, the population density of NC is relatively lower than that of other populations. Whether the worse performance of the NC population is correlated with its genetic background remains unknown and necessitates further investigation. Factors such as geographic isolation and farming patterns often affect the adaptation of populations to the environment and initiate genetic differentiation [51]. The Mantel test showed that there was no significant correlation between genetic distances and geographical distances, indicating that genetic differentiation of these populations is not caused by geographical isolation, but other factors, such as tillage practices and farmland landscape patterns [52].

It has been demonstrated that frequent gene flow can improve the population’s adaptability to the environment and cause outbreaks of pests [53]. In this study, gene flow was found to have existed among *C. oryzae* populations, which was in line with the previous study [30]. However, the degree of gene flow varied remarkably with geographic populations, and it was probably related to the occurrence level of these populations. For instance, QJ and YY populations that showed intensive gene flow performed much better than other populations, while NC and GY populations which showed restricted gene flow occurred lightly.

The genetic structure of *C. oryzae* has been analyzed previously by Zhou et al. [30]. Likewise, we analyzed the genetic structure of *C. oryzae* as well, but using more geographic populations collected from a larger area, within which the ecological environment is more diverse and the annual occurrence generation of *C. oryzae* changes accordingly [27]. Both studies revealed that *C. oryzae* populations have low or medium levels of genetic differentiation and experienced recent expansion events. However, disparities in the genetic diversity and experience of the “Bottleneck effect” were presented between two studies, probably due to the differences in sample size, molecular markers, and sampling locations.

The “Bottleneck effect” refers to the dramatic variation of genetic structure owing to the sharp reduction in population size caused by deteriorated environmental conditions, such as farmland ecology and pesticide application levels [54]. For the past several decades, *C. oryzae* has been subjected to highly toxic pesticides, such as furadan, oxamyl, and triazophos [25]. We, therefore, speculated that the “Bottleneck effect” of *C. oryzae* might be caused by the abundant use of these pesticides. In addition, the recent expansion of *C. oryzae* may be related to changes in factors such as agroecological environments, tillage and cultivation systems, winter temperatures, and control agents [27].

In the future, the investigations of the relationship between the population dynamics of *C. oryzae* and farmland environments and farming practices may elucidate the causes of the “Bottleneck effect”, genetic differentiation, population expansion, and frequent outbreaks, thereby providing a theoretical basis for formulating management strategies.

## 5. Conclusions

This study showed that *C. oryzae* populations suffered from a recent “Bottleneck effect”, followed by a rapid expansion. We also speculated that genetic differentiation and gene flow among populations are responsible for the geographical differences in the occurrence level.

## Figures and Tables

**Table 1:** Genetic diversity of 31 *C. oryzae* populations based on COI sequences.

	n	h	k	Hd	Pi	Tajima’s D	Fu’ Fs
**TY**	24	4	0.250	0.239 ± 0.113	0.0004 ± 0.0002	−1.7325 **	−3.0208 ***
**ZZ**	24	4	0.250	0.239 ± 0.113	0.0004 ± 0.0002	−1.7325	−3.0208 ***
**XT**	24	3	0.243	0.236 ± 0.109	0.0003 ± 0.0002	−1.2023 *	−1.4074 **
**LH**	23	6	0.435	0.395 ± 0.128	0.0006 ± 0.0002	−1.9921 ***	−4.8874 ***
**BG**	20	3	0.637	0.353 ± 0.123	0.0009 ± 0.0003	−0.6594	0.2535
**DT**	20	3	0.200	0.195 ± 0.115	0.0003 ± 0.0002	−1.5128 **	−1.8631 **
**HX**	8	2	0.250	0.250 ± 0.180	0.0004 ± 0.0003	−1.0548	−0.1820
**JC**	20	5	0.400	0.368 ± 0.135	0.0006 ± 0.0002	−1.8679 **	−3.6541 ***
**LQ**	20	4	1.058	0.432 ± 0.126	0.0015 ± 0.0008	−1.7892 **	0.1219
**MG**	20	4	0.579	0.363 ± 0.131	0.0008 ± 0.0003	−1.4084 *	−1.2369 *
**PJ**	20	4	0.300	0.284 ± 0.128	0.0004 ± 0.0002	−1.7233 **	−2.7493 ***
**TJ**	20	2	0.100	0.100 ± 0.088	0.0001 ± 0.0001	−1.1644	−0.8793 *
**TL**	20	2	0.300	0.100 ± 0.088	0.0004 ± 0.0004	−1.7233 **	0.5439
**WQ**	20	4	0.389	0.363 ± 0.131	0.0005 ± 0.0002	−1.4407 *	−2.1353 ***
**YW**	20	3	0.200	0.195 ± 0.115	0.0003 ± 0.0002	−1.5128 **	−1.8631 **
**BZ**	20	2	0.100	0.100 ± 0.088	0.0001 ± 0.0001	−1.1644	−0.8793 *
**DJY**	20	6	0.589	0.447 ± 0.137	0.0008 ± 0.0003	−1.7800 **	−4.0149 ***
**DZ**	20	2	0.189	0.189 ± 0.108	0.0003 ± 0.0002	−0.5916	−0.0966
**GY**	20	3	0.400	0.195 ± 0.115	0.0006 ± 0.0004	−1.8679 ***	−0.6256
**LS**	17	4	1.118	0.596 ± 0.099	0.0016 ± 0.0003	−0.1695	0.0627
**LZ**	21	3	3.724	0.638 ± 0.058	0.0052 ± 0.0006	2.2431	6.1273
**QL**	20	5	0.779	0.368 ± 0.135	0.0011 ± 0.0005	−1.7190 **	−1.7642 **
**YA**	20	5	0.837	0.368 ± 0.135	0.0012 ± 0.0005	−1.2429	−1.5755 *
**ZJ**	20	4	0.389	0.363 ± 0.131	0.0005 ± 0.0002	−1.4407 *	−2.1353 ***
**FL**	20	4	2.963	0.489 ± 0.117	0.0041 ± 0.0010	0.5779	3.1379
**NC**	12	3	3.985	0.621 ± 0.087	0.0055 ± 0.0006	2.0135	4.6229
**PS**	21	4	0.467	0.348 ± 0.128	0.0007 ± 0.0003	−1.6536 **	−1.6755 **
**QJ**	17	4	0.471	0.331 ± 0.143	0.0007 ± 0.0003	−1.8431 **	−1.8636 **
**WZ**	7	1	0.000	0.000 ± 0.000	0.0000 ± 0.0000	N	N
**XS**	20	4	0.389	0.363 ± 0.131	0.0005 ± 0.0002	−1.4407 *	−2.1353 **
**YY**	20	7	0.600	0.521 ± 0.135	0.0008 ± 0.0003	−2.0562 ***	−5.6554 ***
**Total**	598	55	0.854	0.346 ± 0.026	0.0012 ± 0.0001	−2.4784 ***	−3.40 × 10^38^ ***

(n) Number of individuals; (h) number of haplotypes; (Hd) haplotype diversity; (k) average number of nucleotide differences; (Pi) nucleotide diversity; * *p* < 0.10; ** *p* < 0.05; *** *p* < 0.01.

**Table 2:** Genetic diversity of 26 *C. oryzae* populations based on ITS1 sequences.

	n	h	k	Hd	Pi	Tajima’s D	Fu’ Fs
**BG**	23	9	1.375	0.838 ± 0.056	0.0023 ± 0.0004	−0.8686	−4.5472 ***
**DT**	20	5	1.068	0.558 ± 0.114	0.0018 ± 0.0006	−1.1736	−0.9454
**JC**	24	6	1.145	0.500 ± 0.121	0.0019 ± 0.0006	−0.8693	−1.5615
**LQ**	19	8	1.298	0.649 ± 0.108	0.0021 ± 0.0005	−0.2877	−0.5202
**MG**	22	8	1.576	0.818 ± 0.059	0.0026 ± 0.0004	−0.1321	−2.8304 **
**PJ**	15	6	1.562	0.848 ± 0.054	0.0026 ± 0.0003	0.0531	−1.4952
**TJ**	21	5	1.267	0.595 ± 0.108	0.0021 ± 0.0007	−0.7553	−0.4465
**TL**	17	3	1.250	0.588 ± 0.093	0.0021 ± 0.0003	1.1573	1.5858
**WQ**	10	5	2.356	0.800 ± 0.100	0.0039 ± 0.0009	−0.2034	−0.2303
**YW**	14	4	1.077	0.495 ± 0.151	0.0018 ± 0.0009	−1.5407 **	−0.2586
**BZ**	9	4	1.944	0.806 ± 0.089	0.0032 ± 0.0004	1.3055	0.3315
**DJY**	13	7	1.346	0.795 ± 0.109	0.0022 ± 0.0005	−0.5866	−3.6278 ***
**DZ**	12	6	1.636	0.848 ± 0.074	0.0027 ± 0.0004	−0.0430	−1.8724 *
**GY**	10	7	2.911	0.933 ± 0.062	0.0048 ± 0.0006	0.7476	−2.1342 *
**LS**	10	5	1.022	0.756 ± 0.130	0.0017 ± 0.0004	−0.1297	−2.2036 **
**LZ**	11	3	1.055	0.618 ± 0.104	0.0017 ± 0.0003	1.6648	0.6938
**QL**	10	8	2.733	0.956 ± 0.059	0.0045 ± 0.0006	−0.6167	−3.8821 ***
**YA**	10	6	2.244	0.889 ± 0.075	0.0037 ± 0.0006	0.2410	−1.5332
**FL**	17	7	1.779	0.868 ± 0.045	0.0029 ± 0.0005	−0.4858	−1.9303 *
**NC**	13	4	1.385	0.603 ± 0.131	0.0023 ± 0.0010	−1.4646 *	0.1989
**PS**	10	5	1.022	0.667 ± 0.163	0.0017 ± 0.0005	−0.1297	−2.2036 **
**QJ**	7	5	1.619	0.905 ± 0.103	0.0027 ± 0.0005	−0.0398	−2.0192 **
**SZ**	5	2	0.400	0.400 ± 0.237	0.0007 ± 0.0004	−0.8165	0.0902
**WZ**	10	4	0.933	0.533 ± 0.180	0.0015 ± 0.0006	−0.4313	−1.0204
**XS**	17	7	1.360	0.809 ± 0.079	0.0022 ± 0.0004	0.4581	−2.8195 **
**YY**	11	5	1.382	0.618 ± 0.164	0.0023 ± 0.0008	−0.7301	−1.2656 *
**Total**	360	26	1.551	0.750 ± 0.021	0.0026 ± 0.0001	−1.2621 *	−16.0072 ***

(n) Number of individuals; (h) number of haplotypes; (Hd) haplotype diversity; (k) average number of nucleotide differences; (Pi) nucleotide diversity; * *p* < 0.10; ** *p* < 0.05; *** *p* < 0.01.

**Table 3:** Analysis of molecular variance (AMOVA) of COI and ITS1 sequences from the *C. oryzae* populations.

	Source of Variation	*d.f.*	SS	Variance Components	%	F-Statistic
**COI**	Among populations	30	56.336	0.079 Va **	18.32	
	Within populations	567	200.070	0.353 Vb **	81.68	
	Total	597	256.406	0.432	100.00	Fst = 0.183 **
**ITS1**	Among populations	25	35.596	0.050 Va **	6.47	
	Within populations	334	243.645	0.727 Vb **	93.53	
	Total	359	278.383	0.777	100.00	Fst = 0.065 **

(SS) Sum of squares; (%) percentage of variation; (Va) variance components among populations; (Vb) variance components within populations; ** *p* < 0.001.

**Table 4:** Fst (below diagonal) and Nm (above diagonal) between the 31 *C. oryzae* populations based on COI sequences.

	TY	ZZ	XT	LH	BG	DT	HX	JC	LQ	MG	PJ	TJ	TL	WQ	YW	BZ	DJY	DZ	GY	LS	LZ	QL	YA	ZJ	FL	NC	PS	QJ	WZ	XS	YY
**TY**		hun	33.84	hun	84.97	55.43	inf	inf	inf	91.75	inf	10.23	hun	inf	inf	7.53	19.07	1.01	1.96	hun	22.24	inf	30.04	26.64	2.05	0.45	32.27	inf	26.63	hun	inf
**ZZ**	0.00		inf	hun	15.20	55.43	hun	inf	inf	inf	inf	12.80	hun	inf	hun	7.53	19.07	1.01	1.96	hun	22.24	inf	30.04	26.64	2.05	0.45	32.27	inf	26.63	hun	inf
**XT**	0.01	0.00		44.14	10.88	12.64	34.56	34.44	29.49	16.21	32.31	7.35	30.81	32.31	36.43	6.75	14.72	0.97	1.90	36.24	12.62	29.49	19.26	16.21	1.92	0.43	19.09	inf	16.21	39.34	29.77
**LH**	0.00	0.00	0.01		15.60	22.66	inf	inf	inf	hun	inf	8.16	inf	inf	inf	9.38	25.23	1.05	2.37	inf	36.24	inf	43.51	40.25	2.12	0.49	46.32	inf	40.25	hun	hun
**BG**	0.01	0.03	0.04	0.03		hun	56.06	57.78	13.85	22.34	14.99	57.04	inf	14.99	59.38	8.94	14.43	1.84	3.22	17.26	11.92	13.85	15.57	13.73	5.29	0.86	14.20	inf	13.74	19.57	hun
**DT**	0.01	0.01	0.04	0.02	0.00		20.81	inf	16.16	inf	18.48	inf	inf	18.48	43.79	8.35	15.99	1.37	2.57	23.07	12.18	16.16	18.38	15.27	3.17	0.63	16.83	inf	15.28	27.80	inf
**HX**	0.00	0.00	0.01	0.00	0.01	0.02		hun	hun	32.59	hun	7.40	inf	hun	inf	8.96	hun	1.09	2.23	inf	23.21	hun	42.20	hun	2.21	0.50	42.09	inf	32.59	inf	hun
**JC**	0.00	0.00	0.01	0.00	0.01	0.00	0.00		hun	inf	hun	hun	inf	hun	hun	8.96	25.49	1.19	2.23	hun	23.17	hun	42.09	32.57	2.56	0.53	41.98	inf	32.53	hun	inf
**LQ**	0.00	0.00	0.02	0.00	0.03	0.03	0.00	0.00		23.25	hun	5.83	11.98	hun	inf	7.41	20.92	1.04	1.86	hun	13.73	hun	33.01	23.29	2.08	0.46	33.47	inf	23.25	inf	inf
**MG**	0.01	0.00	0.03	0.00	0.02	0.00	0.02	0.00	0.02		hun	37.44	inf	27.93	37.32	8.28	18.40	1.20	2.32	37.18	13.75	23.25	23.17	18.44	2.59	0.55	21.53	inf	18.43	46.85	inf
**PJ**	0.00	0.00	0.02	0.00	0.03	0.03	0.00	0.00	0.00	0.00		6.61	54.09	hun	hun	8.18	23.20	1.08	2.05	hun	18.48	hun	37.55	27.94	2.14	0.48	37.87	inf	27.93	inf	hun
**TJ**	0.05	0.04	0.06	0.06	0.01	0.00	0.06	0.00	0.08	0.01	0.07		16.06	6.61	8.18	5.80	8.36	1.49	2.36	8.18	5.61	5.83	8.28	6.94	3.60	0.67	7.49	hun	6.94	9.74	inf
**TL**	0.00	0.00	0.02	0.00	0.00	0.00	0.00	0.00	0.04	0.00	0.01	0.03		54.09	inf	38.44	inf	1.75	3.64	inf	13.47	11.98	inf	inf	4.24	0.84	inf	inf	inf	inf	inf
**WQ**	0.00	0.00	0.02	0.00	0.03	0.03	0.00	0.00	0.00	0.02	0.00	0.07	0.01		hun	9.73	23.20	1.06	2.05	hun	18.48	hun	37.55	27.94	2.14	0.48	37.87	inf	27.93	inf	inf
**YW**	0.00	0.00	0.01	0.00	0.01	0.01	0.00	0.00	0.00	0.01	0.00	0.06	0.00	0.00		9.73	27.75	1.11	2.42	hun	28.10	inf	hun	37.29	2.27	0.52	46.14	inf	37.32	hun	hun
**BZ**	0.06	0.06	0.07	0.05	0.05	0.06	0.05	0.05	0.06	0.06	0.06	0.08	0.01	0.05	0.05		inf	1.18	hun	hun	6.96	8.80	inf	8.28	2.36	0.59	8.75	inf	8.28	13.39	13.83
**DJY**	0.03	0.03	0.03	0.02	0.03	0.03	0.00	0.02	0.02	0.03	0.02	0.06	0.00	0.02	0.02	0.00		1.20	6.58	inf	15.35	20.92	inf	49.90	2.46	0.59	19.63	inf	18.40	32.35	34.49
**DZ**	0.33 *	0.33 *	0.34 *	0.32 *	0.21	0.27	0.31	0.30	0.32	0.29	0.32	0.25	0.22	0.32	0.31	0.30	0.29		1.18	1.11	1.06	1.04	1.16	1.11	inf	inf	1.10	1.88	1.11	1.16	1.36
**GY**	0.20	0.20	0.21	0.17	0.13	0.16	0.18	0.18	0.21	0.18	0.20	0.17	0.12	0.20	0.17	0.00	0.07	0.30 *		3.99	1.96	1.86	6.24	2.32	2.08	0.64	2.43	4.16	2.32	2.79	2.70
**LS**	0.00	0.00	0.01	0.00	0.03	0.02	0.00	0.00	0.00	0.01	0.00	0.06	0.00	0.00	0.00	0.00	0.00	0.31	0.11		28.02	hun	inf	hun	2.28	0.52	45.97	inf	37.18	hun	hun
**LZ**	0.02	0.02	0.04	0.01	0.04	0.04	0.02	0.02	0.04	0.04	0.03	0.08	0.04	0.03	0.02	0.07	0.03	0.32	0.20	0.02		13.73	18.58	13.77	2.11	0.48	inf	inf	13.75	37.84	24.40
**QL**	0.00	0.00	0.02	0.00	0.03	0.03	0.00	0.00	0.00	0.02	0.00	0.08	0.04	0.00	0.00	0.05	0.02	0.32	0.21	0.00	0.04		33.01	23.29	2.08	0.46	33.47	inf	23.25	inf	80.93
**YA**	0.02	0.02	0.03	0.01	0.03	0.03	0.01	0.01	0.01	0.02	0.01	0.06	0.00	0.01	0.00	0.00	0.00	0.30	0.07	0.00	0.03	0.01		23.15	2.37	0.55	25.77	inf	23.17	55.93	55.93
**ZJ**	0.02	0.02	0.03	0.01	0.04	0.03	0.00	0.02	0.02	0.03	0.02	0.07	0.00	0.02	0.01	0.06	0.01	0.31	0.18	0.00	0.04	0.02	0.02		2.24	0.51	21.54	inf	18.44	46.80	37.58
**FL**	0.20	0.20	0.21	0.19	0.09	0.14	0.18	0.16	0.19	0.16	0.19	0.12	0.11	0.19	0.18	0.17	0.17	0.00	0.19	0.18	0.19	0.19	0.17	0.18		7.37	2.23	5.00	2.24	2.41	3.05
**NC**	0.53	0.53 *	0.54 *	0.51 *	0.37	0.44 *	0.50 *	0.48	0.52	0.48	0.51	0.43 *	0.37	0.51 *	0.49	0.46 *	0.46 *	0.00	0.44 *	0.49 *	0.51 *	0.52	0.47	0.49 *	0.06		0.52	0.88	0.51	0.56	0.63
**PS**	0.02	0.02	0.03	0.01	0.03	0.03	0.01	0.01	0.01	0.02	0.01	0.06	0.00	0.01	0.01	0.05	0.02	0.31	0.17	0.01	0.00	0.01	0.02	0.02	0.18	0.49		inf	21.53	53.73	48.57
**QJ**	0.00	0.00	0.00	0.00	0.00	0.00	0.00	0.00	0.00	0.00	0.00	0.00	0.00	0.00	0.00	0.00	0.00	0.21	0.11	0.00	0.00	0.00	0.00	0.00	0.09	0.36	0.00		inf	inf	inf
**WZ**	0.02	0.02	0.03	0.01	0.04	0.03	0.02	0.02	0.02	0.03	0.02	0.07	0.00	0.02	0.01	0.06	0.03	0.31 *	0.18	0.01	0.04	0.02	0.02	0.03	0.18	0.49	0.02	0.00		46.85	37.64
**XS**	0.00	0.00	0.01	0.00	0.02	0.02	0.00	0.00	0.00	0.01	0.00	0.05	0.00	0.00	0.00	0.04	0.02	0.30 *	0.15	0.00	0.01	0.00	0.01	0.01	0.17	0.47	0.01	0.00	0.01		inf
**YY**	0.00	0.00	0.02	0.00	0.00	0.00	0.00	0.00	0.00	0.00	0.00	0.00	0.00	0.00	0.00	0.03	0.01	0.27	0.16	0.00	0.02	0.01	0.01	0.01	0.14	0.44	0.01	0.00	0.01	0.00	

(inf) Nm is infinite; (hun) Nm > 100; (0.00) Fst ≤ 0.00; * *p* < 0.02; Nm = (1 - Fst)/2Fst.

**Table 5:** Fst (below diagonal) and Nm (above diagonal) between the 26 *C. oryzae* populations based on ITS1 sequences.

	BG	DT	JC	LQ	MG	PJ	TJ	TL	WQ	YW	BZ	DJY	DZ	GY	LS	LZ	QL	YA	FL	PS	NC	QJ	SZ	WZ	XS	YY
**BG**		11.41	inf	6.74	3.29	1.83	inf	7.89	3.58	inf	1.37	0.87	1.80	0.96	12.38	0.75	2.23	1.45	1.72	inf	9.08	5.23	inf	inf	6.63	inf
**DT**	0.02		hun	inf	7.09	2.69	49.45	4.64	3.49	inf	1.28	1.25	3.52	1.01	inf	0.99	2.21	1.65	2.28	inf	3.34	76.44	inf	inf	inf	inf
**JC**	0.00	0.00		7.13	4.94	1.66	23.47	21.04	5.36	inf	1.29	0.84	1.66	1.01	5.09	0.58	2.28	1.48	2.12	61.78	3.58	4.37	inf	inf	4.90	inf
**LQ**	0.04	0.00	0.03		15.24	9.22	8.34	6.28	7.23	17.70	3.35	2.17	hun	1.23	inf	2.25	5.77	3.67	3.57	inf	2.36	inf	inf	inf	inf	inf
**MG**	0.07	0.03	0.05	0.02		inf	2.49	inf	inf	2.71	inf	26.09	inf	5.00	2.68	1.11	inf	inf	inf	5.22	1.32	inf	4.27	3.04	inf	inf
**PJ**	0.12	0.09	0.13	0.03	0.00		1.51	11.42	inf	1.41	inf	inf	inf	5.20	2.43	2.52	inf	inf	inf	3.96	0.90	inf	1.60	1.81	inf	inf
**TJ**	0.00	0.01	0.01	0.03	0.09	0.14		2.57	2.56	inf	1.03	0.81	1.76	0.90	inf	0.90	1.52	1.25	1.29	inf	inf	3.69	inf	inf	8.81	inf
**TL**	0.03	0.05	0.01	0.04	0.00	0.02	0.09		inf	2.61	10.31	2.35	5.70	2.59	1.90	0.66	inf	10.47	inf	4.11	1.22	inf	3.05	2.96	10.68	inf
**WQ**	0.07	0.07	0.04	0.03	0.00	0.00	0.09	0.00		2.30	inf	9.79	inf	inf	2.20	1.06	inf	inf	inf	3.79	1.70	inf	3.76	2.82	15.20	inf
**YW**	0.00	0.00	0.00	0.01	0.08	0.16	0.00	0.09	0.10		0.93	0.76	1.57	0.82	inf	0.71	1.63	1.16	1.39	inf	26.12	3.46	inf	inf	7.00	inf
**BZ**	0.15	0.16	0.16	0.07	0.00	0.00	0.19	0.02	0.00	0.21		inf	inf	inf	1.21	1.35	inf	inf	inf	1.72	0.74	inf	1.01	1.13	6.88	2.90
**DJY**	0.22	0.17	0.23	0.10	0.01	0.00	0.23	0.10	0.02	0.25	0.00		inf	5.61	0.94	1.27	67.32	inf	inf	1.17	0.56	inf	0.71	0.79	4.17	2.22
**DZ**	0.12	0.07	0.13	0.00	0.00	0.00	0.12	0.04	0.00	0.14	0.00	0.00		5.76	3.43	7.33	inf	inf	inf	6.95	1.04	inf	1.90	2.19	inf	76.44
**GY**	0.21	0.20	0.20	0.17	0.05	0.05	0.22	0.09	0.00	0.23	0.00	0.04	0.04		0.89	0.87	83.64	inf	27.16	1.13	0.80	9.52	1.01	0.93	1.75	2.26
**LS**	0.02	0.00	0.05	0.00	0.09	0.09	0.00	0.12	0.10	0.00	0.17	0.21	0.07	0.22		1.81	2.32	1.42	1.42	inf	5.74	8.77	inf	inf	inf	inf
**LZ**	0.25 *	0.20	0.30 *	0.10	0.18	0.09	0.22	0.27	0.19	0.26	0.16	0.16	0.03	0.22	0.12		1.43	1.42	0.94	1.81	0.67	2.63	0.60	0.93	3.57	1.39
**QL**	0.10	0.10	0.10	0.04	0.00	0.00	0.14	0.00	0.00	0.13	0.00	0.00	0.00	0.00	0.10	0.15		inf	inf	3.31	1.11	inf	3.02	2.12	11.24	7.59
**YA**	0.15	0.13	0.14	0.06	0.00	0.00	0.17	0.02	0.00	0.18	0.00	0.00	0.00	0.00	0.15	0.15	0.00		inf	1.97	0.93	inf	1.41	1.36	8.06	4.11
**FL**	0.13	0.10	0.11	0.07	0.00	0.00	0.16	0.00	0.00	0.15	0.00	0.00	0.00	0.01	0.15	0.21	0.00	0.00		2.06	0.85	inf	1.71	1.45	5.86	5.78
**PS**	0.00	0.00	0.00	0.00	0.05	0.06	0.00	0.06	0.06	0.00	0.13	0.18	0.03	0.18	0.00	0.12	0.07	0.11	0.11		8.06	inf	inf	inf	inf	inf
**NC**	0.03	0.07	0.07	0.10	0.16	0.22	0.00	0.17	0.13	0.01	0.25	0.31	0.19	0.24	0.04	0.27	0.18	0.21	0.23	0.03		1.51	inf	inf	2.32	5.38
**QJ**	0.05	0.00	0.05	0.00	0.00	0.00	0.06	0.00	0.00	0.07	0.00	0.00	0.00	0.03	0.03	0.09	0.00	0.00	0.00	0.00	0.14		4.28	5.62	inf	inf
**SZ**	0.00	0.00	0.00	0.00	0.06	0.14	0.00	0.08	0.06	0.00	0.20	0.26	0.12	0.20	0.00	0.29	0.08	0.15	0.13	0.00	0.00	0.06		inf	hun	inf
**WZ**	0.00	0.00	0.00	0.00	0.08	0.12	0.00	0.08	0.08	0.00	0.18	0.24	0.10	0.21	0.00	0.21	0.11	0.15	0.15	0.00	0.00	0.04	0.00		inf	inf
**XS**	0.04	0.00	0.05	0.00	0.00	0.00	0.03	0.02	0.02	0.03	0.04	0.06	0.00	0.13	0.00	0.07	0.02	0.03	0.04	0.00	0.10	0.00	0.00	0.00		inf
**YY**	0.00	0.00	0.00	0.00	0.00	0.02	0.00	0.00	0.00	0.00	0.08	0.10	0.00	0.10	0.00	0.15	0.03	0.06	0.04	0.00	0.04	0.00	0.00	0.00	0.00	

(inf) Nm is infinite; (hun) Nm > 100; (0.00) Fst ≤ 0.00; * *p* < 0.02; Nm = (1 - Fst)/4Fst.

## Data Availability

Sequences used in this study has been deposited in GenBank under accessions number OM490688-OM491162 for COI, and OM540945-OM541304 for ITS1.

## Supplementary Materials

The following supporting information can be downloaded at: https://www.mdpi.com/article/10.3390/insects13040327/s1, Table S1: Information on the *C. oryzae* samples used in this study; Table S2. Geographical distance (km) among *C. oryzae* populations.

## References

[1] Hughes A.R., Inouye B.D., Johnson M.T.J., Underwood N., Vellend M. (2008). Ecological consequences of genetic diversity. Ecol. Lett..

[2] Gienapp P., Teplitsky C., Alho J.S., Mills J.A., Meril J. (2008). Climate change and evolution: Disentangling environmental and genetic responses. Mol. Ecol..

[3] David J.P., Huber K., Failloux A.B., Rey D., Meyran J.C. (2003). The role of environment in shaping the genetic diversity of the subalpine mosquito, *Aedes rusticus* (Diptera, Culicidae). Mol. Ecol..

[4] Huang S., He S.P., Peng Z.G., Zhao K., Zhao E.M. (2007). Molecular phylogeography of endangered sharp–snouted pitviper (Deinagkistrodon acutus; Reptilia, Viperidae) in Mainland China. Mol. Phylogenet. Evol..

[5] Xun H.Z., Li H., Li S.J., Wei S.J., Zhang L.J., Song F., Jiang P., Yang H.L., Han F., Cai W.Z. (2016). Population genetic structure and post–LGM expansion of the plant bug *Nesidiocoris tenuis* (Hemiptera: Miridae) in China. Sci. Rep..

[6] Terhorst C.P., Lau J.A. (2014). Genetic variation in invasive species response to direct and indirect species interactions. Biol. Invasions.

[7] Zheng S.Z., Li Y., Yang X.J., Chen J.Y., Hua J., Gao Y. (2018). DNA barcoding identification of Pseudococcidae (Hemiptera: Coccoidea) using the mitochondrial COI gene. Mitochondrial DNA B Resour..

[8] Fang Y., Shi W.Q., Zhang Y. (2017). Molecular phylogeny of *Anopheles hyrcanus* group members based on ITS2 rDNA. Parasit. Vectors.

[9] Cao L.J., Wang Z.H., Gong Y.J., Zhu L., Hoffmann A.A., Wei S.J. (2017). Low genetic diversity but strong population structure reflects multiple introductions of western flower thrips (Thysanoptera: Thripidae) into China followed by human–mediated spread. Evol. Appl..

[10] Rollins L.A., Woolnough A.P., Sinclair R., Mooney N.J., Sherwin W.B. (2011). Mitochondrial DNA offers unique insights into invasion history of the common starling. Mol. Ecol..

[11] Cameron S.L. (2014). Insect mitochondrial genomics: Implications for evolution and phylogeny. Annu. Rev. Entomol..

[12] Dickey A.M., Kumar V., Hoddle M.S., Funderburk J.E., Morgan J.K., Jara–Cavieres A., Shatters R.G.J., Osborne L.S., McKenzie C.L. (2015). The scirtothrips dorsalis species complex: Endemism and invasion in a global pest. PLoS ONE.

[13] Tyagi K., Kumar V., Singha D., Chandra K., Laskar B.A., Kundu S., Chakraborty R. (2017). DNA Barcoding studies on Thrips in India: Cryptic species and Species complexes. Sci. Rep..

[14] Vissing J. (2019). Paternal comeback in mitochondrial DNA inheritance. Proc. Natl. Acad. Sci. USA.

[15] Dong Z.K., Wang Y.Z., Li C., Li L.L. (2021). Mitochondrial DNA as a molecular marker in insect ecology: Current status and future prospects. Ann. Entomol. Soc. Am..

[16] Triseleva T.A., Petrosyan V.G., Yatsuk A.A., Safonkin A.F. (2020). Morphological and molecular (COI mtDNA) diversity of the polyzonal species of grass flies *Meromyza Nigriseta Fedoseeva*, 1960 (Diptera: Chloropidae). Acta. Zool. Bulg..

[17] Safonkina A.F., Triselevaa T.A., Yatsuka A.A., Petrosyan V.G. (2018). Morphometric and Molecular Diversity of the Holarctic *Meromyza saltatrix* (L., 1761) (Diptera, Chloropidae) in Eurasia. Biol. Bull. Russ. Acad. Sci..

[18] Palomares–Rius J.E., Cantalapiedra–Navarrete C., Archidona–Yuste A., Subbotin S.A., Castillo P. (2017). The utility of mtDNA and rDNA for barcoding and phylogeny of plant–parasitic nematodes from *Longidoridae* (Nematoda, Enoplea). Sci. Rep..

[19] Cruz D.O., Jorge D.M.M., Pereira J.O.P., Torres D.C., Soares C.E.A., Freitas B.M., Grangeiro T.B. (2006). Intraspecific variation in the first internal transcribed spacer (ITS1) of the nuclear ribosomal DNA in *Melipona subnitida* (Hymenoptera, Apidae), an endemic stingless bee from northeastern Brazil. Apidologie.

[20] Pereira J.O.P., Freitas B.M., Jorge D.M.M., Torres D.C., Soares C.E.A., Grangeiro T.B. (2009). Genetic variability in *Melipona quinquefasciata* (Hymenoptera, Apidae, Meliponini) from northeastern Brazil determined using the first internal transcribed spacer (ITS1). Genet. Mol. Res..

[21] Kapantaidaki D.E., Evangelou V.I., Morrison W.R., Leskey T.C., Brodeur J., Milonas P. (2019). *Halyomorpha halys* (Hemiptera: Pentatomidae) Genetic Diversity in North America and Europe. Insects.

[22] Takeda M. (1998). Genetic basis of photoperiodic control of summer and winter diapause in geographic ecotypes of the rice stem maggot, *Chlorops oryzae*. Entomol. Exp. Appl..

[23] Takeda M. (1997). Effects of photoperiod and temperature on larval development and summer diapause in two geographic ecotypes of the rice stem maggot, *C. oryzae* Matsumura (Diptera: Chloropidae). Appl. Entomol. Zool..

[24] Takeda M., Nagata T. (1992). Photoperiodic responses during larval development and diapause of two geographic ecotypes of the rice stem maggot, *Chlorops oryzae*. Entomol. Exp. Appl..

[25] Wang H.D., Xu Z.H., Chen Y.F., Zhu J.X., Fang Y.H. (2007). Rice yield loss due to *Chlorops oryzae* Matsumera and its action thresholds in rice fields in Zhejiang province. Zhi Wu Bao Hu.

[26] Tian P., Qiu L., Zhou A.L., Chen G., He H.L., Ding W.B., Li Y.Z. (2019). Evaluation of appropriate reference genes for investigating gene expression in *C. oryzae* (Diptera: Chloropidae). J. Econ. Entomol..

[27] Tian P., Sun H.M., Chen Y.K., Li X.W., He H.L., Li Y.Z. (2021). Occurrence and fungicides screening of the rice stem maggot *Chlorops oryzae* in Hunan Province. Zhi Wu Bao Hu Xue Bao.

[28] Qiu L., Tao S.J., He H.L., Ding W.B., Li Y.Z. (2018). Transcriptomics reveal the molecular underpinnings of chemosensory proteins in *Chlorops oryzae*. BMC Genom..

[29] Wang J., Li X.Y., Du R.B., Liu Y.H. (2021). The complete mitogenome of *C. oryzae* Matsumura (Diptera: Chloropidae). Mitochondrial DNA B Resour..

[30] Zhou A.L., Tian P., Li Z.C., Li X.W., Tan X.P., Zhang Z.B., Qiu L., He H.L., Ding W.B., Li Y.Z. (2020). Genetic diversity and differentiation of populations of *C. oryzae* (Diptera, Chloropidae). BMC Ecol..

[31] De Barro P.J., Driver F. (1997). Use of RAPD PCR to distinguish the B biotype from other biotypes of *Bemisia tabaci* (Gennadius) (Hemiptera: Aleyrodidae). Aust. J. Entomol..

[32] Yu Y., Pham N., Xia B., Papusha A., Wang G., Yan Z., Peng G., Chen K., Ira G. (2018). Dna2 nuclease deficiency results in large and complex DNA insertions at chromosomal breaks. Nature.

[33] Kumar S., Stecher G., Tamura K. (2016). MEGA7: Molecular evolutionary genetics analysis version 7.0 for bigger datasets. Mol. Biol. Evol..

[34] Librado P., Rozas J. (2009). DnaSP v5: A software for comprehensive analysis of DNA polymorphism data. Bioinformatics.

[35] Fu Y.X. (1997). Statistical tests of neutrality of mutations against population growth, hitchhiking and background selection. Genetics.

[36] Tajima F. (1989). Statistical method for testing the neutral mutation hypothesis by DNA polymorphism. Genetics.

[37] Excoffier L., Lischer H.E. (2010). Arlequin suite ver 3.5: A new series of programs to perform population genetics analyses under Linux and Windows. Mol. Ecol. Resour..

[38] Kantharaja D.C., Lakkundi T.K., Basavanna M., Manjappa S. (2012). Spatial analysis of fluoride concentration in groundwaters of Shivani watershed area, Karnataka state, South India, through geospatial information system. Environ. Earth. Sci..

[39] Rohlf F.J. (2000). NTSYS–pc, Numerical Taxonomy and Multivariate Analysis System, version 2.1e.

[40] Polzin T., Daneshmand S.V. (2003). On Steiner trees and minimum spanning trees in hypergraphs. Oper. Res. Lett..

[41] Amos W., Harwood J. (1998). Factors affecting levels of genetic diversity in natural populations. Philos. Trans. R. Soc. Lond. B Biol. Sci..

[42] Avise J.C. (2010). Perspective: Conservation genetics enters the genomics era. Conserv. Genet..

[43] Toews D.P.L., Brelsford A. (2012). The biogeography of mitochondrial and nuclear discordance in animals. Mol. Ecol..

[44] Fontaine M.C., Snirc A., Frantzis A., Koutrakis E., Ozturk B., Ozturk A.A., Austerlitz F. (2012). History of expansion and anthropogenic collapse in a top marine predator of the Black Sea estimated from genetic data. Proc. Natl. Acad. Sci. USA.

[45] Liao J.C., Jing D.D., Luo G.J., Wang Y., Zhao L.M., Liu N.F. (2016). Comparative phylogeography of Meriones meridianus, Dipus sagitta, and Allactaga sibirica: Potential indicators of the impact of the Qinghai–Tibetan Plateau uplift. Mamm. Biol..

[46] Cristiano M.P., Clemes Cardoso D., Fernandes–Salomão T.M., Heinze J. (2016). Integrating paleodistribution models and phylogeography in the Grass–Cutting Ant *Acromyrmex striatus* (Hymenoptera: Formicidae) in southern lowlands of south America. PLoS ONE.

[47] Vinas J., Bremer J.A., Pla C. (2004). Phylogeography of the Atlantic bonito (*Sarda sarda*) in the northern Mediterranean: The combined effects of historical vicariance, population expansion, secondary invasion, and isolation by distance. Mol. Phylogenet. Evol..

[48] Zhang F.M., Ge S. (2002). Data analysis in population genetics. I. analysis of RAPD data with AMOVA. Sheng Wu Duo Yang Xing.

[49] Rousset F. (1997). Genetic differentiation and estimation of gene flow from F–Statistics under isolation by distance. Genetics.

[50] Miller N.J., Birley A.J., Overall A.D.J., Tatchell1 G.M. (2003). Population genetic structure of the lettuce root aphid, *Pemphigus bursarius* (L.), in relation to geographic distance, gene flow and host plant usage. Heredity.

[51] Slatkin M. (1987). Gene flow and the geographic structure of natural populations. Science.

[52] Fuentes–Contreras E., Basoalto E., Franck P., Lavandero B., Knight A.L., Ramirez C.C. (2014). Measuring local genetic variability in populations of codling moth (Lepidoptera: Tortricidae) across an unmanaged and commercial orchard interface. Environ. Entomol..

[53] Xu Y., Mai J.W., Yu B.J., Hu H.X., Yuan L., Jashenko R., Ji R. (2019). Study on the genetic differentiation of geographic populations of *Calliptamus italicus* (Orthoptera: Acrididae) in sino–kazakh border areas based on mitochondrial COI and COII genes. J. Econ. Entomol..

[54] Nei M., Maruyama T., Chakraborty R. (1975). The bottleneck effect and genetic variability in populations. Evolution.

