# Supplementary material for: Population Genetic Structure of Chlorops oryzae (Diptera, Chloropidae) in China
Source: Insects. 2022 Mar 25;13(4):327. doi: 10.3390/insects13040327 (PMC9032139; doi:10.3390/insects13040327)
Supplement: Supplementary file 1 [file insects-13-00327-s001.zip › Supplementary tables.pdf]

**Table S1**. Information on the *C. oryzae* samples used in this study.

| Populations | Collection sites | Location coordinates | | Altitude (m) | Collection dates |
|---|---|---|---|---|---|
| **TY** | Taoyuan County, Hunan Province | E111.36010 | N28.95753 | 53-78 | 07/8/2018 |
| **ZZ** | Zhuzhou County, Hunan Province | E113.11520 | N27.39123 | 82-117 | 15/8/2018 |
| **XT** | Xiangtan County, Hunan Province | E112.54830 | N27.52307 | 68-146 | 08/8/2018 |
| **LH** | Longhui County, Hunan Province | E110.82483 | N27.46337 | 400-474 | 31/5/2019 |
| **BG** | Baoguan Town, Guiding County, Qiannan Prefecture, Guizhou Province | E107.15560 | N26.26282 | 1240 | 07/6/2020 |
| **DT** | Dongtun Town, Xixiu District, Anshun City, Guizhou Province | E106.25693 | N26.20093 | 1270 | 07/6/2020 |
| **HX** | Chenliang Village, Huaxi District, Guiyang City, Guizhou Province | E106.39100 | N26.27120 | 1096 | 09/6/2019 |
| **JC** | Jichang Town, Xixiu District, Anshun City, Guizhou Province | E106.04754 | N26.11200 | 1300 | 25/5/2020 |
| **LQ** | Linquan Town, Qianxi County, Bijie City, Guizhou Province | E105.86377 | N27.04348 | 1396 | 26/5/2020 |
| **MG** | Mengguan Town, Huaxi District, Guiyang City, Guizhou Province | E106.74333 | N26.39537 | 1120 | 26/5/2020 |
| **PJ** | Panjiang Town, Guiding County, Qiannan Prefecture, Guizhou Province | E107.15350 | N26.45894 | 996 | 05/6/2020 |
| **TJ** | Shizi Town, Pingba District, Anshun City, Guizhou Province | E106.24718 | N26.43928 | 1368 | 05/6/2020 |
| **TL** | Tianlong Town, Pingba District, Anshun City, Guizhou Province | E106.15049 | N26.35402 | 1318 | 05/6/2020 |
| **WQ** | Wenquan Town, Xifeng County, Guiyang City, Guizhou Province | E106.86319 | N27.22047 | 789 | 30/5/2020 |
| **YW** | Yunwu Town, Longli County, Qiannan Prefecture, Guizhou Province | E107.02743 | N26.22985 | 1259 | 09/6/2020 |
| **BZ** | Nanjiang Town, Nanjiang County, Bazhong City, Sichuan Province | E106.87445 | N32.33897 | 1003 | 01/7/2021 |
| **DJY** | Zhongxing Town, Dujiangyan City, Sichuan Province | E103.60046 | N30.92996 | 698 | 28/6/2021 |
| **DZ** | Yongxing Town, Kaijiang County, Dazhou City, Sichuan Province | E107.92288 | N31.14816 | 833 | 02/7/2021 |
| **GY** | Xiasi Town, Jiange County, Guangyuan City, Sichuan Province | E105.19901 | N32.25924 | 903 | 30/6/2021 |
| **LS** | Qiaoba Town, Mabian County, Leshan City, Sichuan Province | E103.60286 | N28.81651 | 878 | 25/6/2021 |
| **LZ** | Deyao Town, Gulin County, Luzhou City, Sichuan Province | E105.70428 | N28.05207 | 723 | 22/6/2021 |
| **QL** | Youzha Town, Qionglai City, Sichuan Province | E103.24654 | N30.40839 | 589 | 28/6/2021 |
| **YA** | Bifengxia Town, Yucheng District, Yaan City, Sichuan Province | E103.00993 | N30.10979 | 909 | 26/6/2021 |
| **ZJ** | Jingning Shezu Autonomous County, Lishui City, Zhejiang Province | E119.63580 | N27.97320 | 300-900 | 05/6/2019 |
| **FL** | Buzi Town, Fuling District, Chongqing City | E107.17288 | N29.60197 | 626 | 03/6/2021 |
| **NC** | Nanping Town, Nanchuan District, Chongqing City | E107.01648 | N29.12798 | 757 | 28/5/2020 |
| **PS** | Changsheng Town, Pengshui County, Chongqing City | E108.31829 | N29.35988 | 752 | 16/6/2021 |
| **QJ** | Apengjiang Town, Qianjiang District, Chongqing City | E108.72461 | N29.15787 | 506 | 18/6/2020 |
| **SZ** | Nanbin Town, Shizhu County, Chongqing City | E108.15697 | N30.00230 | 859 | 17/6/2021 |
| **WZ** | Zhushan Town, Wanzhou District, Chongqing City | E108.25726 | N30.71230 | 632 | 19/6/2021 |
| **XS** | Rongxi Town, Xiushan County, Chongqing City | E108.91851 | N28.50473 | 878 | 19/6/2021 |
| **YY** | Banxi Town, Youyang County, Chongqing City | E108.78840 | N28.76098 | 684 | 02/6/2021 |

**Table S2**. Geographical distance (km) between *C. oryzae* populations.

| | TY | ZZ | XT | LH | BG | DT | HX | JC | LQ | MG | PJ | TJ | TL | WQ | YW | BZ | DJY | DZ | GY | LS | LZ | QL | YA | ZJ | FL | NC | PS | QJ | SZ | WZ | XS | YY |
|---|---|---|---|---|---|---|---|---|---|---|---|---|---|---|---|---|---|---|---|---|---|---|---|---|---|---|---|---|---|---|---|---|
| **TY** | 0 | | | | | | | | | | | | | | | | | | | | | | | | | | | | | | | |
| **ZZ** | 245 | 0 | | | | | | | | | | | | | | | | | | | | | | | | | | | | | | |
| **XT** | 197 | 58 | 0 | | | | | | | | | | | | | | | | | | | | | | | | | | | | | |
| **LH** | 174 | 226 | 170 | 0 | | | | | | | | | | | | | | | | | | | | | | | | | | | | |
| **BG** | 511 | 604 | 553 | 388 | 0 | | | | | | | | | | | | | | | | | | | | | | | | | | | |
| **DT** | 589 | 693 | 641 | 474 | 90 | 0 | | | | | | | | | | | | | | | | | | | | | | | | | | |
| **HX** | 573 | 679 | 626 | 459 | 76 | 15 | 0 | | | | | | | | | | | | | | | | | | | | | | | | | |
| **JC** | 612 | 716 | 664 | 497 | 112 | 23 | 39 | 0 | | | | | | | | | | | | | | | | | | | | | | | | |
| **LQ** | 580 | 718 | 663 | 493 | 155 | 102 | 101 | 105 | 0 | | | | | | | | | | | | | | | | | | | | | | | |
| **MG** | 536 | 641 | 589 | 422 | 44 | 53 | 38 | 76 | 113 | 0 | | | | | | | | | | | | | | | | | | | | | | |
| **PJ** | 499 | 600 | 547 | 381 | 22 | 94 | 79 | 117 | 144 | 41 | 0 | | | | | | | | | | | | | | | | | | | | | |
| **TJ** | 576 | 689 | 636 | 468 | 93 | 27 | 24 | 41 | 77 | 50 | 90 | 0 | | | | | | | | | | | | | | | | | | | | |
| **TL** | 589 | 700 | 647 | 480 | 101 | 20 | 26 | 29 | 82 | 59 | 101 | 14 | 0 | | | | | | | | | | | | | | | | | | | |
| **WQ** | 482 | 618 | 562 | 392 | 110 | 128 | 115 | 148 | 101 | 93 | 89 | 106 | 120 | 0 | | | | | | | | | | | | | | | | | | |
| **YW** | 524 | 618 | 566 | 401 | 13 | 77 | 64 | 99 | 147 | 34 | 28 | 81 | 89 | 111 | 0 | | | | | | | | | | | | | | | | | |
| **BZ** | 570 | 815 | 765 | 662 | 676 | 685 | 676 | 697 | 597 | 661 | 654 | 659 | 669 | 569 | 679 | 0 | | | | | | | | | | | | | | | | |
| **DJY** | 779 | 1004 | 947 | 800 | 624 | 586 | 585 | 587 | 485 | 590 | 606 | 562 | 566 | 520 | 620 | 347 | 0 | | | | | | | | | | | | | | | |
| **DZ** | 411 | 654 | 603 | 497 | 548 | 574 | 562 | 589 | 498 | 541 | 527 | 548 | 560 | 449 | 554 | 165 | 413 | 0 | | | | | | | | | | | | | | |
| **GY** | 694 | 936 | 882 | 760 | 693 | 681 | 676 | 688 | 584 | 669 | 672 | 655 | 663 | 583 | 693 | 158 | 212 | 286 | 0 | | | | | | | | | | | | | |
| **LS** | 755 | 946 | 888 | 724 | 451 | 391 | 395 | 385 | 297 | 410 | 437 | 371 | 371 | 366 | 444 | 501 | 235 | 490 | 412 | 0 | | | | | | | | | | | | |
| **LZ** | 562 | 733 | 676 | 508 | 245 | 213 | 209 | 218 | 113 | 211 | 228 | 187 | 194 | 147 | 241 | 490 | 379 | 406 | 470 | 222 | 0 | | | | | | | | | | | |
| **QL** | 800 | 1017 | 960 | 807 | 599 | 553 | 553 | 551 | 453 | 562 | 582 | 530 | 533 | 500 | 594 | 406 | 67 | 454 | 277 | 180 | 354 | 0 | | | | | | | | | | |
| **YA** | 818 | 1030 | 972 | 816 | 590 | 539 | 540 | 535 | 440 | 552 | 574 | 517 | 519 | 494 | 584 | 443 | 107 | 484 | 317 | 155 | 348 | 40 | 0 | | | | | | | | | |
| **ZJ** | 816 | 645 | 699 | 869 | 1249 | 1338 | 1324 | 1361 | 1361 | 1287 | 1245 | 1334 | 1345 | 1261 | 1262 | 1318 | 1586 | 1186 | 1467 | 1570 | 1367 | 1612 | 1632 | 0 | | | | | | | | |
| **FL** | 412 | 630 | 574 | 429 | 371 | 389 | 378 | 404 | 312 | 359 | 349 | 363 | 375 | 267 | 375 | 306 | 373 | 186 | 350 | 357 | 224 | 389 | 405 | 1227 | 0 | | | | | | | |
| **NC** | 423 | 628 | 570 | 416 | 319 | 334 | 324 | 349 | 258 | 305 | 297 | 308 | 320 | 213 | 322 | 357 | 385 | 241 | 389 | 334 | 175 | 391 | 402 | 1239 | 55 | 0 | | | | | | |
| **PS** | 299 | 518 | 461 | 323 | 363 | 406 | 392 | 425 | 352 | 364 | 342 | 383 | 396 | 277 | 370 | 359 | 486 | 202 | 439 | 462 | 293 | 503 | 519 | 1114 | 114 | 129 | 0 | | | | | |
| **QJ** | 257 | 473 | 416 | 279 | 357 | 409 | 395 | 429 | 366 | 364 | 338 | 388 | 402 | 282 | 366 | 395 | 531 | 234 | 482 | 500 | 319 | 547 | 562 | 1073 | 158 | 166 | 45 | 0 | | | | |
| **SZ** | 331 | 564 | 509 | 384 | 427 | 462 | 450 | 480 | 398 | 424 | 406 | 438 | 451 | 334 | 434 | 287 | 449 | 129 | 377 | 460 | 322 | 474 | 495 | 1139 | 105 | 147 | 73 | 109 | 0 | | | |

| | | | | | | | | | | | | | | | | | | | | | | | | | | | | | | | | |
|---|---|---|---|---|---|---|---|---|---|---|---|---|---|---|---|---|---|---|---|---|---|---|---|---|---|---|---|---|---|---|---|---|
| **WZ** | 357 | 599 | 547 | 439 | 506 | 538 | 526 | 555 | 470 | 502 | 485 | 514 | 526 | 411 | 513 | 223 | 445 | 58 | 337 | 496 | 386 | 481 | 508 | 1144 | 162 | 213 | 150 | 179 | 80 | 0 | | |
| **XS** | 243 | 430 | 373 | 220 | 304 | 367 | 352 | 389 | 342 | 318 | 286 | 350 | 363 | 247 | 314 | 469 | 580 | 309 | 549 | 520 | 319 | 588 | 600 | 1051 | 209 | 198 | 112 | 75 | 182 | 254 | 0 | |
| **YY** | 251 | 451 | 393 | 246 | 321 | 379 | 364 | 400 | 345 | 331 | 302 | 360 | 373 | 255 | 331 | 438 | 555 | 278 | 519 | 505 | 312 | 566 | 579 | 1065 | 183 | 177 | 81 | 45 | 151 | 223 | 31 | 0 |
